# The decision on the embryo to transfer after Preimplantation Genetic Diagnosis for X-autosome reciprocal translocation in male carrier

**DOI:** 10.1186/s13039-018-0409-x

**Published:** 2018-12-29

**Authors:** Sandrine Chamayou, Maria Sicali, Debora Lombardo, Carmelita Alecci, Antonino Guglielmino

**Affiliations:** Unità di Medicina della Riproduzione - Centro HERA, via Barriera del Bosco n 51/53 95030 Sant Agata Li Battiati, Catania, Italy

**Keywords:** Genetic counseling, Preimplantation genetic diagnosis, Reciprocal translocation, X-autosome Translocation, X inactivation

## Abstract

**Background:**

The aim of Preimplantation Genetic Diagnosis (PGD) on embryos produced *in vitro* is to identify the embryos without genetic or chromosomal defect from those embryos that will develop the genetic disease or are chromosomally abnormal. In case of PGD for structural chromosome indication (PGR-SR), the normal/balanced embryos are transferred in the maternal uterus. This protocol is valid and widely applied for autosomal chromosome translocation. But which embryo should be transferred after preimplantation genetic diagnosis (PGD-SR) for X-3 reciprocal translocation in male patient?

**Case presentation:**

The female patient was 26 years old with normal 46,XX karyotype. The male patient had a karyotype with balanced translocation 46,Y,t(X;3)(p11.2;p14)mat, inherited from the mother. The female patient underwent two cycles of ovarian stimulation. In the first cycle, the metaphase II oocytes were vitrified, while in the second cycle they were used as fresh. ICSI was performed on vitrified/warmed and fresh oocytes. Embryos were biopsied at blastocyst stage. Chromosomal analysis was performed by Next Generation Sequencing.

Eleven blastocysts were biopsied from 23 vitrified/warmed and fresh metaphase II oocytes. Two embryos were diagnosed 46,XY; two embryos were diagnosed 46,XX; four embryos were diagnosed with unbalanced translocations and three embryos were diagnosed aneuploid. We knew that the two embryos diagnosed as 46,XX inherited the balanced translocation from the father and the two embryos diagnosed as 46,XY had a normal karyotype. It was explain to the couple that the phenotype of balanced translocated female embryos cannot be predicted because of the random inactivation of X chromosome and that could also occur on the der(X). The couple asked to have a 46,XY embryo transferred. Clinical pregnancy was obtained and non invasive prenatal test confirmed PGD-SR result.

**Conclusions:**

Proposing PGD-SR for gonosome-autosome reciprocal translocation implies the risk to exclude balanced translocated female embryos with a normal phenotype for transfer because the early and late normal development at post-natal stage cannot be predicted based on the only chromosomal analysis.

## Background

The primary aim of genetic/chromosomal investigation on embryos produced in vitro is to identify the embryos without genetic/chromosomal defect from those embryos that will develop the genetic disease or are chromosomally unbalanced. The embryos without genetic disease or chromosomally balanced are chosen for embryo-transfer. Preimplantation genetic diagnosis (PGD) is applied in couples with a high risk of inherited defect on offspring such as chromosomal rearrangements including balanced translocations, inversions or deletions [1]. In balanced (Robertsonian or reciprocal) translocations, the carrier parent contains the correct amount of genetic material. If the translocations did not occur within a gene, there is no consequence on carrier phenotype. Nevertheless, the translocated patient has an increased risk for chromosomally unbalanced gametes and conceptions. In case of PGD for structural chromosomal indication (PGD-SR), the embryo diagnosed as diploid for the two entire chromosomes involved in the translocation is chosen for transfer. With the use of the last advanced molecular technologies such as Comparative Genome Hybridization array [2], Single Nucleotide Polymorphism arrays with full molecular karyotyping [3, 4], and Next Generation Sequencing (NGS) [5, 6], a comprehensive chromosomal screening can be performed with a resolution inferior to 1Mb [7, 8]. In routine protocols, these methods do not distinguish an embryo with a normal from an embryo with a balanced translocated karyotype. They remain quantitative on the opposite to banding karyotype.

Among chromosomal translocations, reciprocal translocations that are the product of material exchange between two non-homologous chromosomes are reported with an incidence about 1 in 625 newborns [9]. In particular, balanced gonosome-autosome translocations are very rare, with an incidence estimated in about 1/30,000 live births [10]. Actually, the only available databases that we are aware of on PGD are those from the ESHRE PGD Consortium. On 5247 PGD-SR, only 42 cases due to female or male indications were recorded [11]. In particular, there were listed 16 cases for X-autosome translocation with female indication, 24 cases for Y-autosome translocation with male indication and only 2 cases for X-autosome translocation with male indication [12].

In 1962, Mary Lyon established that one X-chromosome is randomly inactivated in woman to equilibrate the X-linked gene expression between the two sexes [13]. In women with a balanced X-autosome translocation, the normal X chromosome is preferentially inactivated [14] and a normal phenotype is expressed. Nevertheless, the choice of the X chromosome to inactivate is random and can occur on the der(X). In this case, the gene silencing is spread into the autosomal attached segment leading to female patients with genetic disorders with variable gravity.

We presently describe a case of PGD-SR for reciprocal X-3 (p11.2;p14) translocation in male patient. Normal male and balanced translocated female embryos were obtained. We discuss the dilemma of choosing of the embryo to transfer according to sex, in those embryos with unpredictable phenotype. The present work is the first published case of PGD-SR for gonosome-autosome translocation with male indication.

## Case presentation

### Material and methods

#### Patients

The female patient was 26 years old at the time of PGD-SR. Karyotype was 46,XX. Basal FSH, LH on day 3 and AMH value were respectively 8.0 IU/l, 6.5 IU/l and 6.3 ng/ml. She had a 30 days ovarian cycle. Uterine cavity and fallopian tubes resulted regular from diagnostic examination. The ovaries had a polycystic ovarian aspect. The patient had no previous pregnancy.

**Fig. 1:**
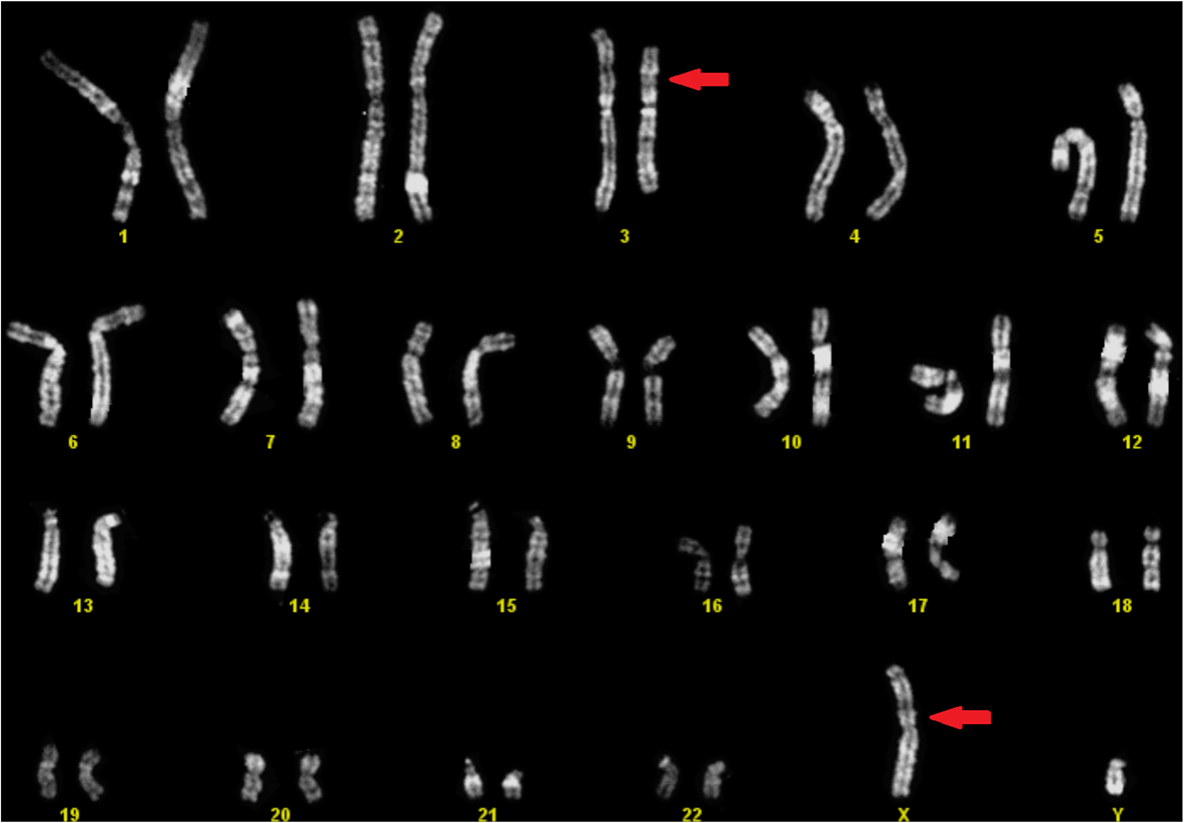
Karyotype 46,Y,t(X,3)(p11.2;p14)mat of male patient

The male patient was 30 years old at the time of PGD-SR. After QFQ-banding karyotype analysis, he was found to have a karyotype with balanced translocation involving X chromosome and autosome 3: 46,Y,t(X;3)(p11.2;p14)mat (Fig. 1).

**Fig. 2:**
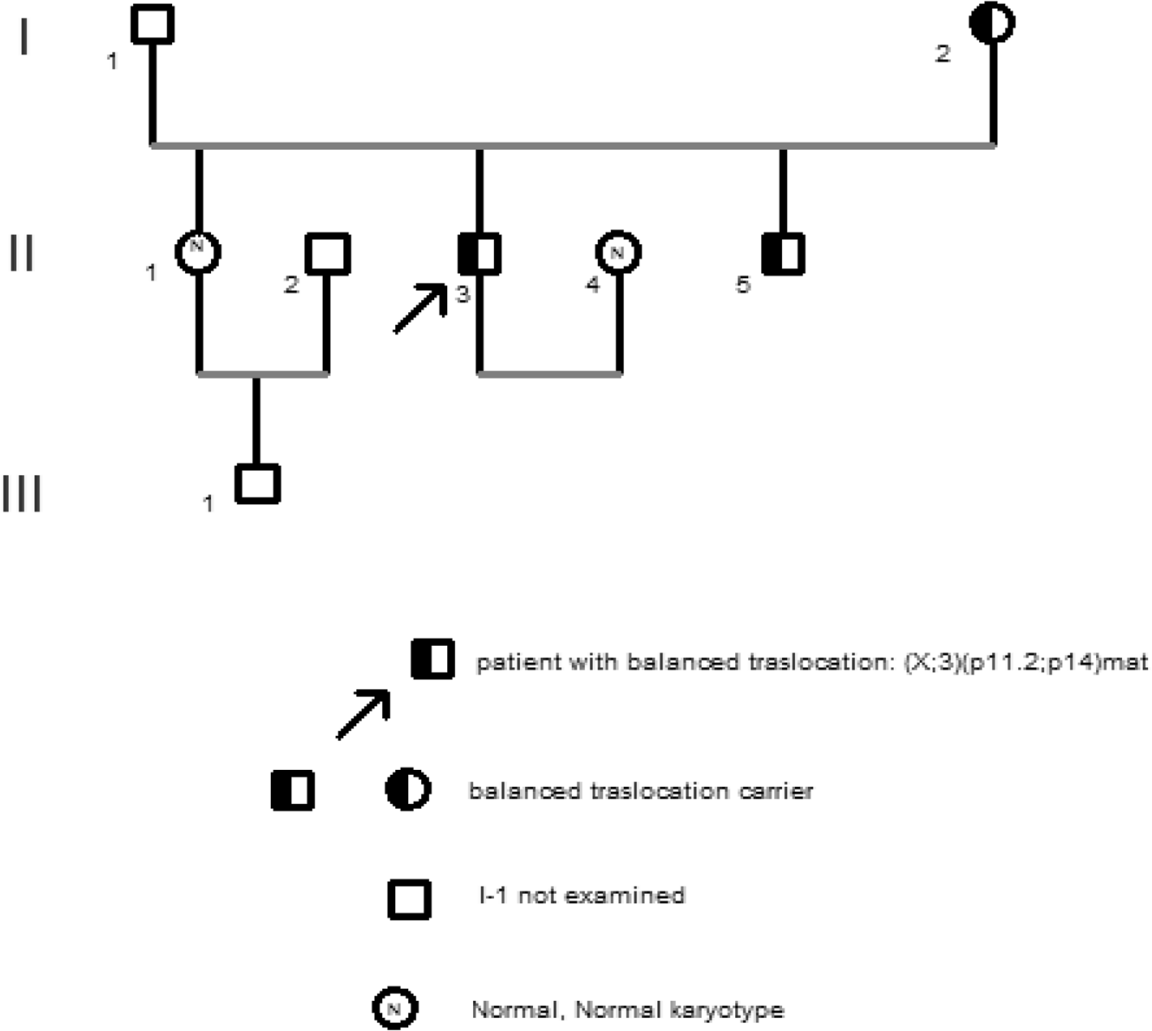
Pedigree of the male's patient family

The X-3 reciprocal translocation was transmitted from the mother that had the karyotype 46,X,t(X;3)(p11.2;p14) in the blood cells. In the family of male patient, the first sibling had a 46,XX normal karyotype (II-1). The third of three siblings (II-5) showed the same karyotype 46,Y,t(X;3)(p11.2;p14)mat of the patient. The parents of the patient were not consanguineous (Fig. 2). His mother and siblings had a normal phenotype.

The male patient had a normal phenotype with normal genital tract.

Semen was characterized by severe oligoasthenoteratozoospermia (Semen concentration: 20.000 sperm /ml; 5% sperm with progressive motility, 2% of normal sperm). From blood cells analysis, the male patient resulted negative for microdeletions of AZFa, AZFb and AZFc regions [15]. The male endocrine was normal for FSH, LH, total Testosterone, Free Testosterone, 17bE2, Prolactin, HCG, aFP. The couple consulted for the first time an infertility center for primary infertility and asked to know 'the state of health' of the embryos as allowed by the Italian law on Medically Assisted Procreation [16].

The following protocols of ovarian stimulation, oocyte and embryo vitrification, ICSI and embryos culture, embryo biopsy and NGS have been described elsewhere and are resumed as follow [17]. Each part of the protocol has been approved by the Institutional Review Board Unità di Medicina della Riproduzione - Centro HERA. The patients signed informed consent forms on all procedures prior application.

#### Ovarian stimulation

The female patient underwent two cycles of ovarian stimulation. In the first cycle, the metaphase II oocytes were vitrified. In the second cycle the metaphase II oocytes were used as fresh together with the previously vitrified/warmed oocytes.

The first ovarian stimulation was performed by the administration of recombinant FSH and LH (Puregon, MSD, Franklin Lakes, USA and Luveris: Merck-Serono, London, UK) from cycle day 2 in a luteal gonadotrophin-releasing hormone antagonist flexible schema (Orgalutran : MDS). Initial doses were 200 IU/day for FSH and 75 IU/day for LH. Luteal gonadotrophin-releasing hormone antagonist was given when the leader follicle reached 14 mm in diameter with a dosage of 0.25 mg/day. The second ovarian stimulation started on day 2 of the following cycle.

One ICSI session was performed using the accumulated vitrified/warmed oocytes together with the lately produced fresh oocytes and fresh male patient semen.

#### ICSI on vitrified/warmed and fresh oocytes and embryo culture

Vaginal ultrasound-guided aspiration of oocyte−cumulus complex (OPU) was performed 35 hours after human chorionic gonadotrophin administration (HCG 10,000 IU, Gonasi: AMSA, Rome, Italy). ICSI was performed on fresh oocytes 3h after OPU and on cryopreserved oocytes 1 hour after warming and in vitro culture with the same patient's fresh ejaculated spermatozoa sample.

After ICSI, in vitro culture was carried out in Continuous single culture complete medium with human serum albumin (Irvine Scientific, Santa Ana, USA) under mineral oil and in automated incubators with 5% CO2, 5% O2 at 37°C, fitted with time-lapse imaging acquisition (Embryoscope, Unisense, Aarhus, Denmark).

#### Embryo biopsy

Embryo biopsies were performed on day 5 on expanded or hatching blastocysts. Few trophectoderm cells [5 to 10] were removed from a zona pellucida hole using a 1.48 um diode laser (OCTAX, Bruckberg, Germany) and a 20 um inner diameter biopsy pipette. After the biopsy procedure, each embryo was incubated until embryo vitrification and before blastocyst re-expension. The biopsied trophectoderm cells were washed in sterile phosphate buffered saline (PBS) solution and transferred into a 0.2 ml Eppendorf tube containing 4 ul of sterile PBS solution.

#### Oocyte/embryo vitrification and warming

The vitrification and warming protocols for oocyte [18] and embryos [19] were previously described.

#### Cell lysis, whole genome amplification and NGS protocol

The biopsied trophectoderm cells were submitted to alkaline lyses and whole genome amplification according to Repli-g Single Cell protocol (Qiagen, Hilden, Germany). After quantification of amplified DNA, libraries were prepared from 100 ng of each sample and barcoded with IonXpressPlus Fragment and IonXpress Barcode Adapter kits (Life Technologies-Thermo Fisher (Carlsbad, USA). After quantification of the libraries, normalization to 100 pM and mix-up to obtain a final concentration of 8 pM, the eleven enriched libraries were loaded on Chip 16 V2. DNA sequencing was performed on ION PGM HiQ View Sequencing in Ion Personal Genome Machine. The updated Torrent Suite Software was used for base calling and mapping on human genome reference sequence Hg19. For each chromosome read coverage was corrected by guanine-cytosine calculation. Aneuploidy was diagnosed comparing data to baseline values multiple male samples. In all the process, a positive control with normal male DNA and a negative control from biopsy culture media were processed together with the samples to diagnose. Genetic analysis was validated when median absolute pair wise difference (MAPD) was inferior to 0.3. Chromosomal segments as short as 7 Mb could be detected. The protocol was previously validated on single cells from amniocytes with different karyotypes [17].

#### Endometrial preparation for embryo transfer

Warming and single embryo transfer was performed on natural cycle at 7 days after LH surge.

### Results

#### *In vitro* results

The results of oocyte vitrification, ICSI, embryo culture and embryo biopsy analysis from vitrified/warmed and fresh oocytes are presented in Table 1.

**Table 1:** in vitro results of PGD-SR treatment

	Vitrified/warmed oocytes (1st ovarian stimulation)	Fresh oocytes (2nd ovarian stimulation)
Metaphase II oocytes at OPU	8	16
Vitrified oocytes	8	-
Survived oocytes	7	-
Micro-injected oocytes	7	16
Zygotes (fertilization rate)	5 (71.4)	12 (75.0)
Expanded/hatching biopsied Blastocysts (proportion on zygote)	3 (60.0)	8 (67.0)
Vitrified biopsied blastocysts	3	8

The patient produced 8 metaphase II oocytes that were vitrified during the first ovarian cycles and 16 metaphase II oocytes in the second ovarian cycle that were used as fresh. Seven of the 8 vitrified oocytes survived to warming and were micro-injected together with the 16 fresh oocytes with the male patient’ semen sample and in the same ICSI procedure. The fertilization rates were 71.4% for vitrified/warmed oocytes (5 zygotes/7 micro-injected oocytes) and 75.0% for fresh oocytes (12 zygotes/16 micro-injected oocytes). On day V, 3 expanded or hatching blastocysts from vitrified/warmed oocytes (3 blastocysts/5 zygotes, 60.0%) and 8 expanded or hatching blastocysts from fresh oocytes (8 blastocysts/12 zygotes, 67.0%) were biopsied and vitrified.

#### Genetic results

The genetic analysis were validated and completed respectively in 100% of the biopsied blastocysts from vitrified/warmed and fresh oocytes. Chromosome contents of each blastocyst after PGD-SR are reported in Table 2.

**Table 2:** Chromosome content of blastocyst after PGD-SR

Embryo n.	Oocyte origin	Chromosome content	n. Reads	Coverage	MAPD
1	Fresh	Trisomy 3p14→cen→3qter and monosomy Xp11.2→cen→Xqter	130,326	99.90%	0.170
3	Fresh	46,XY	106,478	99.91%	0.217
4	Fresh	Trisomy 3p14→cen→3qter and monosomy Xp11.2→cen→Xqter	278,023	99.78%	0.176
6	Fresh	Trisomy 3p14→cen→3qter and monosomy Xp11.2→cen→Xqter	90,688	99.92%	0.226
12	Fresh	46,X,t(X;3)(Xqter→Xp11.2::3p14→3pter;3qter➔3p14::Xp11.2➔Xpter)pat	70,759	99.95%	0.226
13	Fresh	46,XY	124,119	99.90%	0.203
14	Fresh	45,X0	125,875	99.90%	0.198
16	Fresh	45,XY,del [2]	412,006	99.66%	0.153
17	Vitrified/warmed	46,XY,del [8](qter →q22.1)	157,459	99.87%	0.176
18	Vitrified/warmed	Trisomy 3p14→cen→3qter and monosomy Xp11.2→cen→Xqter	118,589	99.90%	0.200
22	Vitrified/warmed	46,X,t(X;3)(Xqter→Xp11.2::3p14→3pter;3qter➔3p14::Xp11.2➔Xpter)pat	133,527	99.89%	0.179

*MAPD* median absolute pair wise difference. Coverage:percentage of bases in whole genome covered by at least 20% of the average base coverage depth reads

**Fig. 3:**
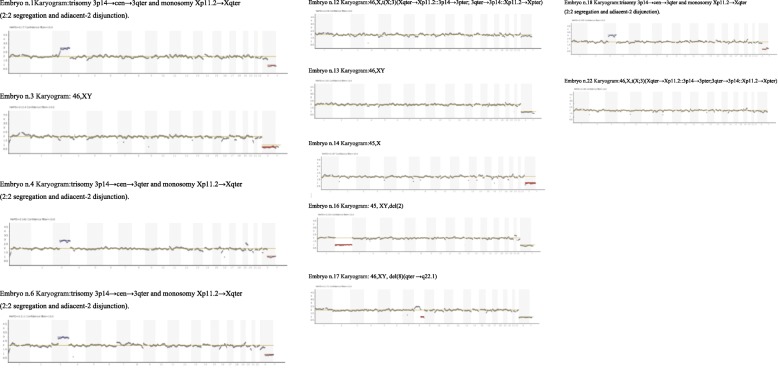
Karyograms of diagnosed embryos.

According to the karyograms generated by IGV (Integrative Genomics Viewer), it was found that two embryos were diagnosed 46,XY, two embryos were diagnosed 46,XX; four embryos were diagnosed with unbalanced translocations and showed the trisomy 3p14→cen→3qter and monosomy Xp11.2→cen→Xqter, from 2:2 segregation and adjacent-2 disjunction. Three embryos were diagnosed aneuploid (45,X0 ; 45,XY,del [2] ;46,XY,del [8](qter →q22.1). Even if balanced translocation could be detected by NGS analysis, the 2 embryos diagnosed as 46,XX were known to have the balanced translocation inherited by the father and their true karyotype was: 46,X,t(X;3)(Xqter→Xp11.2::3p14→3pter;3qter➔3p14::Xp11.2➔Xpter)pat. On the same way, the 2 embryos diagnosed as 46,XY had a normal karyotype. Karyograms are shown in Fig. 3.

#### Genetic counseling

Prior to PGT-SR, a genetic counselling was performed and the couple was informed on the possible karyotypes due to father reciprocal translocation: embryo(s) with normal 46,XY, female(s) with balanced translocation and unpredictable phenotype, embryo(s) with unpredictable complete or partial aneuploidies.

After PGT-SR, the patients were informed on the result of the present PGD-SR. It was explained that among the viable embryos, the 2 male embryos had the true kayotype 46,XY, 2 female embryos had the reciprocal translocation 46,X,t(X;3)(Xqter→Xp11.2::3p14→3pter;3qter➔3p14::Xp11.2➔Xpter)pat.

Regarding the 2 female embryos with balanced translocation, it was explained that the phenotype should be normal, due to the preferable silencing of normal X as it seems to have happened in the patient’s mother. Consequently, deciding not to transfer balanced translocated female means taking the risk of eliminating from transfer and pregnancy a viable embryo with a normal phenotype.

On the other side, genetic disorders with different degree of gravity (from gonadal digenesis and premature ovarian failure to major genetic disorders and mental retardation) were reported in female patients with balanced reciprocal X-autosome translocations. This risk exists and remains unpredictable. The probability of occurring cannot be calculated.

Finally, one embryo had 45,X0 karyotype. It was explained that Turner Syndrome has a large expressivity [20] and a very high in uterus lethality within the first trimester of pregnancy [21, 22].

It was reminded that de novo balanced translocation could not be detected by the present protocol of NGS.

After counselling, the couple asked to have one 46,XY embryo thawed and transferred.

#### Clinical outcome

The embryo n. 13 obtained from fresh oocyte, was thawed and transferred. The β-HCG test performed 12 days after embryo transfer was positive. Two weeks later the clinical pregnancy was ascertained by scan of embryonic sac and one foetal heart beat was observed. Non invasive prenatal testing (NIPT) for all autosome and gonosome chromosomes was performed on the 15th week of pregnancy and confirmed PGD-SR result.

## Discussion and conclusions

We reported a rare case of PGD-SR for reciprocal X-3 translocation in male patient. The couple came for primary infertility and the male patient discovered to have the karyotype 46,XY,t(X;3)(p11.2;p14)mat in diagnostic phase. The reciprocal translocation was inherited from his mother that had a normal phenotype. The male patient had a severe oligoasthenoteratozoospermia probably due to the structural chromosome abnormality [23]. ICSI was proposed to address the male infertility due to sperm parameters alteration and the couple asked to know ‘the state of health of their embryos’ as allowed by the Italian law [16]. Consequently, PGD-SR protocol for complete and partial aneuploidie for all chromosomes was applied. On the 11 embryos obtained after two ovarian stimulations and from vitrified/warmed and fresh oocytes, four embryos (36,4%) were diagnosed with a normal karyotype after NGS analysis, two 46,XY and two 46,XX embryos. Even if it was not possible to detect it by NGS, we knew that the two 46,XY embryos had a normal karyotype (excepted if de novo balanced translocations occurred), and the two 46,XX embryos carried the reciprocal translocation inherited from the father and were 46,X,t(X;3)(Xqter→Xp11.2::3p14→3pter;3qter➔3p14::Xp11.2➔Xpter)pat.

In 2016, Ferfouri et al. presented two cases in which the female patient carried a balanced reciprocal translocation X-autosome [24]. After embryo biopsy on day 3 and FISH analysis, 2/30 (6.7%) embryos resulted normal/balanced. No live birth was obtained after the embryo transfer of two balanced female embryos. In the present case, 4/11 (36.4%) were analysed as chromosomally balanced after embryo biopsy at blastocyst stage.

Among the gonosome-autosome translocations, the X-autosome reciprocal translocations are known to be the most sensitive cases because the phenotype can vary in female carriers. In these cases the normal X chromosome is preferentially inactivated forming the Barr body [25] and the phenotype is normal. Nevertheless, the phenomenon of X inactivation controlled by the X-inactivation centre (XIC) located in Xq13 occurs randomly in one chromosome or the other. In the present case and if lyonisation occurs in the translocated X chromosome, there will be monosomy of autosomic's segment translocated in X chromosome happens on the der(X)t(3pter→3p14::Xp11.2→Xpter) containing Xq13, the der(3)t(Xpter →Xp11.2::3p14→3qter) will not be silenced. Consequently, there will be a double dosage of genes from the portion (Xpter→Xp11.2) and a monosomy of the portion (3pter→3p14).

It has been previously reported that balanced X-autosome translocated females can express an unpredictable phenotype that can vary from gonadal dygenesis and premature ovarian failure to multiple congenital anomalies and mental retardation [26].

In human the X inactivation starts on day 3 due to the silencing process of one X chromosome in female embryos. In the present PGD-SR application, embryo biopsy was performed on day 5 on trophectoderm cells. These cells will generate embryonic annexes and not be part of the embryo. No published data has shown that the study of gene silencing in trophectoderm cells would be representative of gene silencing in the balanced translocated female embryo itself. One of the few alternatives to predict the phenotype of female infants could be the study of X-chromosome inactivation/DNA methylation from foetus blood cells, in prenatal phase [27]. But once again, it remains to demonstrate that X-chromosome inactivation from foetus blood cells would be truly representative of overall gene expression in the infant, the girl and the woman [28].

Regarding the request of the couple to know the state of health of their embryos, we could not answer *in sensus strictus* on the early and late development of the balanced translocated female embryos at post-natal stage as précised during the pre-PGT-SR genetic counselling. At the post-PGT-SR genetic counselling, the couple decided not to consider them as the first choice for embryo transfer. This choice opens an ethical dilemma because, at the same time, embryos with a probably normal phenotype are eliminated from the transfer and possible birth. This would have happened to the male patients’ mother at her time! The couple asked to have one 46,XY embryo transferred. They obtained the pregnancy and PGD-SR was confirmed by NIPT.

Among those embryos with abnormal karyotype, there was a 45,X0 embryo with a potential to implant but high probability to miscarriage. The couple rejected its transfer in uterus. There were 6 non-viable embryos left. One embryo with 45,XY,del [2], one embryo with 46,XY,del [8](qter →q22.1) and four embryos with trisomy 3p14→cen→3qter and monosomy Xp11.2→cen→Xqter. The last four embryos were the product of fertilization from a normal haploid 23,X oocyte and an unbalanced 23,der(X)t(X;3)(Xpter→Xp11.2::3p14→3qter) spermatozoon. These spermatozoa were the product of adjacent-2 chromosome segregation of the translocated chromosomes during the first meiotic division. This unbalanced segregation is reported as the most rare in reciprocal translocation [29]. Similar chromosomal abnormalities were observed in embryos from vitrified/warmed (embryos n. 18 and 22) and fresh oocytes (embryos n. 1, 4, 6 and 12). None of the analysed embryos showed mosaicism.

The present case is the first PGD-SR for gonosome-autosome translocation with male indication. Van Echten-Arends et al. previously reported a PGD-SR gonosome-autosome translocation with female indication that resulted in misdiagnosis for technical problem [30]. Comprehensive chromosomal screening by NGS is the method of choice for PGD of structural chromosomal defect.

This present PGD-SR application for reciprocal X-3 translocation in male patient arises the dilemma of balanced embryos excluded for transfer due to their phenotype unpredictability. The present case shows the limits of PGD-SR in helping the couple with a gonosome-autosome translocation to have a healthy child. Other PGD cases are well-known to be unsolvable at preimplantation stage due to the impossibility to predetermine the phenotype and development of the conceptus. Some of them are PGD for rare CFTR mutations and unknown combined phenotype, multifactory disease (heart defects, cancers, diabetes,…), diseases with variable expressivity (Marfan Syndrome, hereditary non-polyposis colorectal cancer,…) and diseases with late onset (ex: Alzheimer’s disease). In these cases, proposing PGD implies the elimination of possible normal embryos for transfer.

## Abbreviations

aFP: alpha feto protein
AMH: Anti-Müllerian hormone
AZF: Azoospermic Factor
CFTR: Cystic Fibrosis Transmembrane conductance Regulator
FISH: Fluorescent In Situ hybridization
FSH: Follicle-Stimulating Hormone
HCG: Human chorionic gonadotropin
ICSI: Intra Cytoplasmic Sperm Injection
LH: Luteinizing Hormone
MAPD: Median Absolute Pair wise Difference
NGS: Next Generation Sequencing
NIPT: Non Invasive Prenatal Testing
OPU: Oocyte Pick-UP
PGD: Preimplantation Genetic Diagnosis
PGD-SR: Preimplantation Genetic Diagnosis for Structural chromosomal indication
